# Clinical presentation and genetic variants in patients with autoinflammatory diseases: results from the German GARROD registry

**DOI:** 10.1007/s00296-023-05443-x

**Published:** 2023-09-25

**Authors:** Norbert Blank, Ina Kötter, Marc Schmalzing, Jürgen Rech, Karoline Krause, Birgit Köhler, Dorothee Kaudewitz, Martin Nitschke, Christian S. Haas, Hanns-Martin Lorenz, Martin Krusche

**Affiliations:** 1 Department of Internal Medicine V, Division of Rheumatology and Amyloidosis Center, University of Heidelberg, Im Neuenheimer Feld 410, 69120 Heidelberg, Germany; 2 Zentrum Für Seltene Erkrankungen Heidelberg (ZSE HD), Heidelberg, Germany; 3 Department of Rheumatology, University Hospital Eppendorf, Martinistraße 52, 20251 Hamburg, Germany; 4 Clinic for Rheumatology and Immunology, Bad Bramstedt, Germany; 5 Department of Internal Medicine II, Rheumatology and Clinical Immunology, University of Würzburg, Oberdürrbacherstraße 6, 97080 Würzburg, Germany; 6 Friedrich-Alexander-University Erlangen-Nürnberg (FAU), Department of Internal Medicine 3-Rheumatology and Immunology, Universitätsklinikum Erlangen, Erlangen, Germany; 7 Deutsches Zentrum Immuntherapie, Friedrich-Alexander-Universität Erlangen-Nürnberg and Universitätsklinikum Erlangen, 91054 Erlangen, Germany; 8 Zentrum Für Seltene Erkrankungen Erlangen (ZSE ER), Erlangen, Germany; 9 Department of Dermatology, Charité-Campus Mitte, Luisenstraße 2, 10117 Berlin, Germany; 10 Städtisches Klinikum Karlsruhe, Department of Internal Medicine I, Nephrology and Rheumatology, Moltkestraße 90, 76133 Karlsruhe, Germany; 11 Division of Nephrology, Internal Medicine I, Universityhospital S.-H. Campus Lübeck, Ratzeburger Allee 160, 23562 Lübeck, Germany; 12 University of Marburg, Department of Internal Medicine, Nephrology and Intensive Care Medicine, Baldingerstrasse 1, 35033 Marburg, Germany; 13 Department of Rheumatology and Clinical Immunology, Charité University Hospital, Charitéplatz 1, 10117 Berlin, Germany; 14 Internal Medicine 5, Amyloidosis Center and Division of Hematology, Oncology and Rheumatology, University Hospital Heidelberg, Im Neuenheimer Feld 410, 69120 Heidelberg, Germany

**Keywords:** Anakinra, Canakinumab, Familial Mediterranean fever, Cryopyrin-associated periodic syndrome, Tumor necrosis factor receptor-associated periodic syndrome, Mevalonate kinase deficiency

## Abstract

To investigate clinical symptoms and genetic variants in patients from the German anti-IL-1 registry for autoinflammatory orphan diseases (GARROD) between 2013 and 2022. Multicentre, retrospective analysis of demographic, clinical and genetic data of patients with autoinflammatory diseases (AID) who received anti-IL-1 targeted therapy. The cohort comprised 152 patients with familial Mediterranean fever (FMF; n = 71), cryopyrin-associated periodic syndromes (CAPS; n = 43), TNF-receptor associated periodic syndrome (TRAPS; n = 19), mevalonate kinase deficiency (MKD; n = 3) and unclassified AID (uAID; n = 16). Inflammatory attacks started in 61.2% of the patients before the age of 18 years. The delay between the first AID attack and anti-IL-1 therapy was 17.8 years. Monogenetic AIDs were diagnosed by clinical symptoms. Genetic analyses confirmed the diagnosis in 87.3% of patients with FMF, 65.2% with CAPS and 94.8% with TRAPS. Among this group, heterozygous *MEFV* variants and variants of unknown significance (VUS) were detected in 22.5% of patients with FMF, 51.2% with CAPS and 47.4% with TRAPS. Patients with VUS were older at disease onset which is consistent with a milder phenotype. Twenty-four patients had secondary AA amyloidosis (AA) at initiation of anti-IL-1 therapy. The mean age of these patients was 16.4 years at their first attack and 44.9 years at the time of AA diagnosis. Turkish-Armenian ancestry correlated with *MEFV* variants and higher FMF disease activity compared to German ancestry. Molecular genetic analyses should substantiate the clinical diagnosis of a monogenetic AID. Our data support the concept of variable penetrance of VUS which can be associated with late-onset AID.

**Supplementary Information:**

The online version contains supplementary material available at 10.1007/s00296-023-05443-x.

## Introduction

The increasing knowledge about AID has changed our view of systemic inflammation over the recent decades. FMF is the most prevalent AID worldwide. Diagnostic criteria were developed for the monogenetic AIDs familial Mediterranean fever (FMF) [1] and Cryopyrin-associated periodic syndrome (CAPS) [2]. In addition, provisional classification criteria for Tumor necrosis factor receptor-associated periodic syndrome (TRAPS) [3, 4] and mevalonate kinase deficiency (MKD) have been proposed [3, 4]. Although genetic variants for FMF, CAPS, TRAPS and MKD are known, these variants are neither essential for AID diagnosis nor have they been included in the diagnostic criteria. Furthermore, patients with unclassified AID (uAID) show clinical features of classical AID but do not meet diagnostic AID criteria [4, 5]. Genetic analyses in uAID patients do not show pathogenic variants in the majority of patients [5].

Investigations on the pathophysiology of fever led to the recognition of interleukin-(IL)-1α and IL-1β which are two non-redundant proinflammatory cytokines. The discovery of the inflammasome led to the focus on IL-1β as the key cytokine in AID. Blocking of IL-1, either with the IL1-receptor antagonist anakinra (ANA) or with monoclonal antibody canakinumab (CAN), are effective treatment options for AID [6–8].

The German Anti-IL1 treatment RegistRy in Orphan Diseases (GARROD) was initiated in 2013 when it became evident that blocking IL-1 is effective in AID. The GARROD registry is a multicentric retrospective analysis of adult patients with AID which were treated with ANA or CAN on an individual basis.

We present data from a retrospective analysis of a national multi-centre cohort of patients with AID who received IL-1 inhibiting treatment. The primary aim was to analyse the genotype–phenotype correlation and the prevalence of AA amyloidosis in our cohort.

## Material and methods

### Patient data

The GARROD registry was established in October 2013 as a multi-centre registry comprising a consecutive cohort of adult patients with AID who were treated with ANA or CAN for at least two months. The purpose of the registry was to gather and analyse diagnoses, safety and efficacy of IL-1 blocking treatment in patients with AID. Inclusion criteria were the diagnosis of an autoinflammatory syndrome, the persistence of an anti-IL1-targeted therapy for at least 3 months and a partial or complete response to the therapy. Exclusion criteria were patients with minimal or no response to anti-IL1-therapy and patients who were lost to follow-up within the first three months of treatment. The study was approved by the ethical review committee at the University of Heidelberg (S-103/2013) and by the local ethical committees at the participating centres. Until December 2022 various institutions in Germany contributed depersonalized data to the registry.

#### Clinical diagnoses of AID and genetic analysis

FMF [1], CAPS [2], TRAPS [3], MKD [4] and unclassified AID (uAID) [5] were diagnosed by AID experts, which was consistent with the previously published criteria [1–5]. Patients with the clinical diagnosis of a monogenetic AID were screened for variants in *MEFV* (FMF), *NLRP3* (CAPS), *TNFRSF1A* (TRAPS) and *MVK* (MKD). Genetic analyses were performed by Sanger analysis and were conducted at several commercial laboratories. Patients with uAID did not show any pathogenic variants in the mentioned genes. Advanced techniques like multi-panel gene testing and whole exome sequencing were performed recently in selected cases but were not included in this analysis.

Single nucleotide polymorphisms (SNP) with a minor allele frequency (MAF) of ≥ 1% in the general population were considered as a variant of unknown significance (VUS) or as a benign variant. The Infevers database [9, last access 04 Aug 2023] served as a reference to evaluate the functional role of single variants. The nomenclature of genetic variants followed the current guidelines of the Human Genome Organisation (HUGO), Human Genome Nomenclature Committee (HGNC), Human Genome Variation Society (HGVS) and the Infevers database [9]. The MAF was derived from the National Center for Biotechnology Information (NCBI) SNP database (https://www.ncbi.nlm.nih.gov/pubmed).

#### FMF activity and definition of colchicine resistance

FMF activity was assessed by using the Pras score [10] and the ISSF score [11]. The Pras score indicates mild (3–5), moderate (6–9) or severe FMF activity (10–19) [10]. The ISSF score indicates mild (0–2), intermediate (3–5) or severe (6–10) FMF disease activity [11]. The colchicine dose was titrated according to the clinical tolerance with a preferred dose of 2 mg per day. Colchicine-resistant FMF (crFMF) was defined by more than three typical FMF attacks per year despite the regular use of colchicine prophylaxis [12]. Patients with FMF and AA amyloidosis and subclinical elevated CRP and SAA were also considered for IL-1 targeted inhibition treatment as an add on to colchicine which is consistent with FMF treatment recommendations [13]. As FMF patients with kidney transplants cannot take colchicine due to the interaction with calcineurin inhibitors used to prevent rejection of the transplant kidney these patients were also considered for anti-IL-1 treatment with ANA or CAN.

#### Anti-IL-1 targeted medication

ANA was initiated using 100 mg sc daily. Patients with renal failure started with ANA 100 mg sc three times a week. Patients with severe local reactions to ANA used either topical treatment of the injection sites or transiently reduced their schedule to ANA injections every two to three days until local reactions disappeared. Patients with partial control of the attacks used ANA 100 mg twice daily during an attack and 100 mg every day for maintenance.

CAN was initiated using 150 mg sc every 8 weeks. Patients with a partial response reduced the intervals and increased the dose up to CAN 300 mg every 4 weeks to maximize the response and to achieve remission.

Patients who had an inadequate response or intolerance to ANA were switched to CAN.

#### Concomitant medication

Concomitant medication comprised colchicine, pulses of steroids and disease modifying anti-rheumatic drugs (DMARDs) where applicable. Concomitant medication was continued if the patient reported an additional efficacy compared with IL-1 inhibition monotherapy. FMF patients continued with colchicine if they tolerated at least 0.5 mg per day or more. The majority of FMF patients (> 90%) received 2 mg colchicine per day. Patients with uAID had concomitant colchicine if they reported any partial response before the addition of IL-1 inhibition. Patients with CAPS, TRAPS, MKD and uAID who had three or fewer attacks per year used pulses of prednisolone 40 mg and tapering or ANA on-demand to control the attacks.

#### Diagnosis of amyloidosis

All patients were screened for renal impairment, proteinuria and myocardial hypertrophy as potential indicators for amyloidosis [14]. If functional organ abnormalities were detected, the affected organs were biopsied. The diagnosis of amyloidosis was based on a positive Congo red staining and a yellow-green birefringence on polarization microscopy. The type of AA amyloid was confirmed by immunohistochemistry [15].

#### Statistics

Statistical analyses were performed using the Statistical Package for the Social Sciences (IBM SPSS Version 19) and the R statistical program (The R Foundation for Statistical Computing Platform, version 3.3.2.). A Kolmogorov–Smirnov test was used to confirm normality in the distribution of numeric variables. Results are presented as mean and standard deviation (SD), median and interquartile range (IQR) or range as indicated. Categorical variables were compared using Fishers exact test. Numerical variables were compared with unpaired, two-tailed Student’s T-test. Statistical significance is considered if p was below 0.05.

## Results

One hundred and fifty-two adult patients with AID were analysed in the GARROD registry. Usually, NSAIDs, colchicine or glucocorticoids failed to control the systemic inflammation. Therefore, inhibition of IL-1 treatment was initiated and patients were enrolled in the GARROD registry immediately or within the following 12 months. Demographic characteristics are displayed in Table 1. The mean age at first AID attack varied between 11.7 years in MKD and 27.5 years in uAID. The disease duration between the first AID attack and the initiation of anti-IL-1 targeted therapy which corresponded to the recruitment to the GARROD registry at visit-1 varied between 12.4 years in TRAPS and 24.3 years in FMF (Table 1).

**Table 1:** Demographic parameters of 152 patients with AID at the initiation of an anti-IL1 targeted therapy

Total cohort n = 152	FMF	CAPS	TRAPS	MKD	uAID
n	71	43	19	3	16
Female gender n (%)	40 (56.3)	32 (74.4)	13 (68.4)	2 (66.7)	12 (75.0)
Age at first AID attack					
Mean ± SD (years)	15.2 ± 16.1	20.3 ± 19.0	21.6 ± 13.5	11.7 ± 17.8	27.5 ± 19.8
p	0.019	0.765	0.21	0.644	0.072
Age at visit-1					
Mean ± SD (years)	39.5 ± 13.7	41.4 ± 15.2	34.1 ± 12.2	33.1 ± 7.9	44.8 ± 13.9
p	0.79	0.075	0.285	0.78	0.148
Disease duration at visit-1					
Mean ± SD (years)	24.3 ± 14.1	21.2 ± 15.5	12.5 ± 10.1	21.4 ± 14.8	17.3 ± 16.6
p	0.013	0.029	0.004	0.686	0.331

### AID attacks in children and adults with monogenetic and unclassified AID

FMF (52.2%, n = 71), CAPS (31.6%, n = 43) and TRAPS (14.0%, n = 19) were the most prevalent diagnoses in our cohort (Table 2). The majority of adult patients with AID (61.2%; n = 93) reported that their first AID attack occurred in childhood or adolescence. However, 38.8% of the adult patients (n = 59) reported that their first AID attack occurred after the age 18 years (Table 2). Patients with uAID showed a trend for a higher age at onset (56.2% after age 18 years) indicating a milder phenotype in the absence of a pathogenic genotype.

**Table 2:** Adult patients with monogenetic and undifferentiated AID report the onset of AID attacks before or after age 18 years

	Total n (%)	Age at first AID attack		p
		Onset < 18y	Onset ≥ 18y	
Monogenetic AID	136 (100)	86 (63.2)	50 (36.8)	0.059
Mean ± SD		7.4 ± 5.4	35.8 ± 14.9	0.072
FMF n (%)	71 (52.2)	49 (69.0)	22 (31.0)	
CAPS n (%)	43 (31.6)	24 (55.8)	19 (44.2)	
TRAPS n (%)	19 (14.0)	11 (57.9)	8 (42.1)	
MKD n (%)	3 (2.2)	2 (66.7)	1 (33.3)	
Unclassified AID	16 (100)	6 (37.5)	10 (62.5)	Comparator
Mean ± SD		9.3 ± 4.5	38.4 ± 17.0	Reference
Total	152	93 (61.2)	59 (38.8)	

### Pathogenic variants correlate with an early-onset of AID attacks

Molecular genetic analyses were performed in 131 of 136 (96.3%) patients with a clinical diagnosis of a monogenetic AID. The results of genetic variants in FMF (*MEFV*), CAPS (*NLRP3*), TRAPS (*TNFRSF1A*) and MKD (*MVK*) and their biological function according to the Infevers homepage are shown in the supplemental table S1 (recessive trait AID) and supplemental table S2 (dominant trait AID).

Genetic variants were considered to be pathogenic (p), a variant of unknown significance (v), a benign polymorphism or wild type (w) (Table 3). The clinical diagnosis of FMF was genetically confirmed with two pathogenic *MEFV* variants, either homozygous or compound heterozygous, in 64.8% of patients with FMF. The rate of molecular genetic confirmation increased to 87.3% if *MEFV* variants of unknown significance or heterozygous *MEFV* variants (pv, pw, vv or vw) were considered to contribute to the phenotype (Table 3). Nine patients with a clinical FMF diagnosis (12.7%) had no *MEFV* variant in exome 1–10.

**Table 3:** The genotype phenotype correlation in 136 patients with monogenetic AID

Trait monogenetic AID	Recessive FMF	Dominant CAPS	Dominant TRAPS	Recessive MKD	n (%)
*Gene*	*MEFV*	*NLRP3*	*TNFRSF1A*	*MVK*	
Patients (n)	71	43	19	3	136
Genotype confirmed	pp	pw	pw	pp	
n (%)	45 (63.4)	6 (14.0)	9 (47.4)	2 (66.7)	62 (45.6)
Age at first attack (y)	10.8 ± 13.4	6.2 ± 4.4	17.0 ± 12.1	2.0 ± 1.4	
p	reference	reference	reference	reference	
Genotype variant	pv + pw + vv + vw	vv + vw	vv + vw	pw	48 (35.3)
n (%)	16 (22.5)	22 (51.2)	9 (47.4)	1 (33.3)	
Age at first attack (y)	22.8 ± 18.2	19.6 ± 14.7	26.3 ± 14.6	32	
p	0.025	0.001	0.16	n.a	
Genotype wild type	ww	ww	ww	ww	21 (15.4)
n (%)	9 (12.7)	12 (27.9)	0	0	
Age at first attack (y)	24.2 ± 18.3	29.0 ± 26.8	–	–	
p	0.063	0.014	–	–	
Genotype unknown	1 (1.4)	3 (7.0)	1 (5.3)	0	5 (3.7)

Variants were classified as pathogenic (p), variant of unknown significance (v) or wildtype (w) according to [9]. A T-test was used for numeric variables

CAPS and TRAPS are rare diseases with an autosomal dominant trait. Pathogenic variants were confirmed in 14.0% of *NLRP3* and 47.4% of *TNFRSF1A* analyses. The detection of VUS (51.2% in *NLRP3*, 47.4% in *TNFRSF1A,* Table 3) supported the clinical diagnosis of a monogenetic AID and the initiation of an IL-1 blocking therapy. High penetrance variants were associated with a younger age at the first AID attack. Low penetrance variants or VUS were associated with a more variable clinical course and a higher age at the first AID attack as reported by the patients (Table 3). MKD was diagnosed in three non-related patients from families with a Turkish, Italian and German background. Patients with FMF had a predominant Turkish-Armenian ancestry. Patients with CAPS and TRAPS had a predominant German origin.

### Pathogenic MEFV variants correlate with a higher FMF severity score and with AA amyloidosis

FMF was the most abundant AID in this cohort. The *MEFV* variants p.Met680Ile/Val and p.Met694Val/Ile are known as pathogenic variants with high penetrance (HP). Homozygous or compound heterozygous MEFV variants (n = 2) are associated with a more severe FMF disease. Patients with two HP variants were younger at FMF onset (mean age 11.3 years vs. 15.9 years (one HP) vs 21.2 years (no HP), had a higher FMF activity score (mean Pras 9.5 vs 8.5 (one HP) vs. 6.8 (no HP) and were more much more likely to develop secondary AA amyloidosis (Table 4). Patients with FMF + AA had a mean age of 17.3 years at FMF onset and 45.6 years at the diagnosis of AA amyloidosis (Table 5). Patients reported an AID disease activity over three decades until the diagnosis of AA amyloidosis was established. HP variants were more prevalent in patients with AA amyloidosis (18 versus 6) when compared to non-AA patients with AID (44 versus 63; p = 0.0032).

**Table 4:** High penetrance *MEFV* variants correlate with FMF severity

High penetrance *MEFV* variants	2	1	0	p 2 vs 0
Patient number (n)	35	15	21	
Age at first FMF attack (mean ± SD)	11.3 ± 15.0	15.9 ± 15.4	21.2 ± 17.2	0.0300
Pras FMF activity score (mean ± SD)	9.5 ± 5.4	8.5 ± 3.1	6.8 ± 3.9	0.0008
ISSF score (mean ± SD)	4.7 ± 1.9	3.3 ± 1.7	2.5 ± 1.5	0.00001
Fever n (%)	30 (85.7)	12 (80.0)	18 (85.7)	1.0000
Peritonitis n (%)	32 (91.4)	13 (86.7)	10 (47.6)	0.0004
Pleuritis n (%)	22 (62.9)	11 (73.3)	10 (47.6)	0.2820
Leg pain n (%)	30 (85.7)	11 (73.3)	16 (76.2)	0.4760
Arthritis n (%)	11 (31.4)	5 (33.3)	5 (23.8)	0.7610
AA amyloidosis n (%)	16 (45.7)	3 (20.0)	3 (14.3)	0.0207
Turkish-Armenian ancestry n (%)	35 (100.0)	15 (100.0)	10 (47.6)	
German ancestry n (%)	0	0	11 (52.4)	0.00001

**Table 5:** HP variants are more prevalent in patients with monogenetic AID and AA amyloidosis

	n (%)	Genotype	Age at fist AID attack	Age at AA diagnosis	Disease duration mean ± SD (years)
Patients with AA amyloidosis	24 (15.8)		16.4 ± 18.4	44.9 ± 11.1	28.6 ± 13.1
AA and FMF	22 (31.0)		17.3 ± 18.9	45.6 ± 11.4	28.3 ± 13.7
	16	2xHP	17.2 ± 19.9	45.4 ± 12.5	28.2 ± 13.4
	3	1xHP	23.7 ± 25.4	46.7 ± 6.0	23.0 ± 20.2
	3	0xHP	11.7 ± 7.4	45.7 ± 12.9	34.0 ± 10.8
AA and TRAPS	1 (5.3)	1xHP	9	42	33
AA and MKD	1 (33.3)	2xHP	3	16	13

### Ancestry correlated with MEFV genotype and FMF disease activity

FMF patients with Turkish-Armenian ancestry more likely carried high penetrance (HP) *MEFV* variants and had significantly higher disease activity scores (Pras; p = 0.0050; ISSF p < 0.00001; Table 6). All the patients with German ancestry and a clinical FMF diagnosis had at least one *MEFV* variant but no HP *MEFV* variants were detected in patients with German ancestry. The partial response to colchicine, fever, pleuritis and exertional leg pain was not different when compared to FMF patients of Turkish-Armenian ancestry (Table 6). Peritonitis and AA amyloidosis seemed to be less prevalent, but differences were not statistically significant in these small subgroups.

**Table 6:** FMF patients with German ancestry are heterozygous for *MEFV* variants and have a milder FMF disease activity

FMF ancestry	Turkish-Armenian (n = 60)		p	German (n = 11)	
AA amyloidosis	No	Yes		No	Yes
n (%)	37 (62)	23 (38)	0.0847	10 (91)	1 (9)
Age at FMF mean ± SD	12.5 ± 12.1	15.3 ± 17.3	0.5756	20.6 ± 18.8	8
Age at AA mean ± SD	n.a	37.2 ± 13.3		n.a	48
Colchicine (mg/d)	1.7 ± 0.8	1.5 ± 0.6	0.3595	1.5 ± 0.4	2.0
Pras score mean ± SD	8.1 ± 2.9	10.3 ± 2.9	0.0050	6.6 ± 2.3	8
ISSF score mean ± SD	3.0 ± 1.2	6.0 ± 1.2	0.0000	2.2 ± 0.8	5
Fever; n (%)	32 (87)	20 (87)	1.0000	8 (80)	0
Peritonitis; n (%)	33 (89)	19 (83)	0.4684	3 (30)	0
Pleuritis; n (%)	22 (60)	15 (65)	0.7866	6 (60)	0
Leg pain; n (%)	31 (84)	19 (83)	1.0000	7 (70)	0
Arthritis; n (%)	15 (41)	5 (22)	0.1662	1 (10)	0
Any *MEFV*; n (%)	32 (86)	23 (100)	0.1460	9 (90)	1 (100)
2xHP *MEFV*; n (%)	17 (46)	18 (78)	0.0168	0	0
*SAA1α/α*; n (%)	4/24 (17)	9/18 (50)	0.0410	0	1 (100)

## Discussion

This is a multicentre retrospective analysis of 152 patients with the monogenetic AID FMF, CAPS, TRAPS and MKD and unclassified AID.

Even though this is a relatively small cohort compared with the large FMF cohorts from Israel [10] and Turkey [16] one has to bear in mind that Germany is a low-prevalence country for FMF. However, all our patients were treated with anti-IL-1 targeted biologics which indicates that our study is focused on patients with high disease activity and represents a selection of more severe disease within a disease spectrum.

Our data show that AID attacks can develop in adults in a significant proportion of patients. When we perform genetic analyses, we have to interpret the results and correlate the genetic variants with the clinical phenotype. Especially the interpretation of genetic variants of unknown significance (VUS) is difficult. Our results indicate that pathogenic variants are associated with an earlier onset of AID attacks and VUS are related to a later onset in FMF, CAPS and TRAPS.

Although the Tel Hashomer FMF criteria [1] were developed in a country with a high prevalence of pathogenic MEFV variants, we identified patients with a German ancestry who also met the diagnostic criteria, partially responded to colchicine, carried at least one *MEFV* variant and responded to anti-IL1 targeted therapy. The FMF disease activity scores were significantly higher in patients with Turkish-Armenian ancestry and lower in Germans. Interestingly, typical attacks of peritonitis which are a hallmark in the majority of FMF patients seem to be less frequent in FMF patients of German ancestry. Other features like recurrent febrile attacks, pleuritis and leg pain were similarly prevalent in FMF patients of Turkish and German ancestry. FMF patients who can tolerate colchicine should add anti-IL1 targeted therapy according to current EULAR recommendations [13].

It remains an open question whether genetic confirmation is necessary and sufficient to confirm a clinical AID diagnosis and whether VUS contribute to an AID phenotype. Some authors suggested that patients without a clear genetic confirmation of the clinical diagnosis and especially patients with VUS should be considered as uAID [5]. The syndrome of undifferentiated recurrent fever (SURF) was suggested by paediatric rheumatologists to describe a heterogeneous group of AID characterized by self-limiting episodes of systemic inflammation of unknown origin without a confirmed molecular diagnosis and not fulfilling the criteria for periodic fever, aphthous stomatitis, pharyngitis and adenopathy (PFAPA) syndrome [17]. Nevertheless, since patients with SURF often show a complete or partial response to colchicine treatment [18] they might also meet the clinical criteria for FMF [1]. PFAPA is common in children and adolescents but rare in adults. The pathogenesis of PFAPA remains elusive and no genetic variants were consistently associated with PFAPA. From a scientific point of view, a genetic confirmation of AID should be the goal. The contribution of VUS to an AID phenotype should be supported by functional experimental analyses [19].

The presence of 0, 1 or 2 high penetrance *MEFV* variants (M680I, M694I or M694V) in our cohort inversely correlated with the age at onset, and directly correlated with FMF activity and the presence of AA amyloidosis. These data were consistent with a gene dose–response of *MEFV* variants that have been shown by others recently [20, 21].

The relevance of variants of unknown significance (VUS) remains to be uncertain in all monogenetic AID. The *MEFV* variant E148Q is considered to be associated with a milder FMF disease if other variants are present [19, 22]. Patients with homozygous E148Q/E148Q variants can have a milder disease or remain asymptomatic [23]. The *NLRP3* variants V198M and Q703K are considered as VUS or benign polymorphism by some authors [9, 24, 25] and as low penetrance variants by others [26, 27, 28]. The most prevalent *TNFRSF1A* variant R92Q is considered as VUS by some authors [9] and a low penetrance variant by others [29, 30]. Recently, whole exome sequencing and targeted deep sequencing were used to screen for pathogenic variants in other genes or somatic variants which might contribute to an autoinflammatory phenotype in selected patients [31, 32]. The low prevalence of AA amyloidosis in patients with CAPS and TRAPS might be associated with the relatively low disease activity which is characterized by the late-onset of inflammatory attacks in patients with VUS.

Limitations of our study were the retrospective design and a partial dependence on the patient’s recall of their first attacks. Another limitation of the study was the lack of whole exome sequencing in patients with unclassified AID. Therefore, rare pathogenic variants in other genes might have been missed.

Our cohort study analysed the clinical presentation, disease severity, molecular genetic results and treatment of AID. Molecular genetic analyses should substantiate the clinical diagnosis of a monogenetic AID. Our data support the concept of variable penetrance of VUS which can be associated with late-onset AID. The delay between onset of AID, diagnosis and treatment must be shortened to prevent AA amyloidosis and irreversible organ damage.

### Supplementary Information

Below is the link to the electronic supplementary material.

**Supplemental Table 1:** Autosomal recessive diseases: *MEFV *variants in FMF and *MVK *variants in MKD with minor allele frequencies and biologic function according to [9]. **Supplemental Table 2:** Autosomal dominant diseases: *NLRP3 *variants in CAPS and *TNFRSF1A *variants in TRAPS with minor allele frequencies and biologic function according to [9] (DOCX 21 KB)

## Data Availability

Genetic variants of MEFV and MVK are indicated in Supplemental Table 1. Genetic variants of NLRP3 and TNFRSF1A are indicated in Supplemental Table 2. Further data are available upon request via the corresponding author.

## Abbreviations

AA: Amyloid-A amyloidosis
AID: Autoinflammatory disease
ANA: Anakinra
CAN: Canakinumab
CAPS: Cryopyrin-associated periodic syndrome
crFMF: Colchicine refractory FMF
FMF: Familial Mediterranean fever
GARROD: German Anti-IL1 treatment RegistRy in Orphan Diseases
MKD: Mevalonate kinase deficiency
p: Pathogenic variant
SURF: Syndrome of undifferentiated fever
TRAPS: Tumor necrosis factor receptor-associated periodic syndrome
uAID: Unclassified AID
v orVUS: Variant of unknown significance
w: Wild type genotype
