# Supplementary material for: Clinical presentation and genetic variants in patients with autoinflammatory diseases: results from the German GARROD registry
Source: Rheumatol Int. 2023 Sep 25;44(2):263–71. doi: 10.1007/s00296-023-05443-x (PMC10796580; doi:10.1007/s00296-023-05443-x)
Supplement: Supplementary file 1 — Supplemental Table 1: Autosomal recessive diseases: MEFV variants in FMF and MVK variants in MKD with minor allele frequencies and biologic function according to [9]. Supplemental Table 2: Autosomal dominant diseases: NLRP3 variants in CAPS and TNFRSF1A variants in TRAPS with minor allele frequencies and biologic function according to [9] (DOCX 21 KB) [file 296_2023_5443_MOESM1_ESM.docx]

**Supplemental Table 1**

| **Diagnosis** | **Gene** | **Exon** | **HGVS protein** | **SNP** | **RefSNP Report** | **MAF** | **Function** |
| --- | --- | --- | --- | --- | --- | --- | --- |
| FMF | *MEFV* | 5 | p.Phe479Leu | F479L | rs104895083 | 0.00001 | pathogenic |
| FMF | *MEFV* | 10 | p.Met680Ile | M680I | rs28940580 | 0.00006 | pathogenic |
| FMF | *MEFV* | 10 | p.Met694Val | M694V | rs61752717 | 0.002 | pathogenic |
| FMF | *MEFV* | 10 | p.Lys695Arg | K695R | rs104895094 | 0.002 | pathogenic |
| FMF | *MEFV* | 10 | p.Val726Ala | V726A | rs28940579 | 0.0002 | pathogenic |
| FMF | *MEFV* | 10 | p.Arg761His | R761H | rs104895097 | 0.0002 | pathogenic |
| FMF | *MEFV* | 2 | p.Glu148Gln | E148Q | rs3743930 | 0.126 | VUS |
| FMF | *MEFV* | 2 | p.Gln230Lys | E230K | rs104895080 | 0.00001 | VUS |
| FMF | *MEFV* | 3 | p.Pro369Ser | P369S | rs11466023 | 0.02 | VUS |
| FMF | *MEFV* | 3 | p.Arg408Gln | R408Q | rs11466024 | 0.007 | VUS |
| FMF | *MEFV* | 9 | p.Ile591Thr | I591T | rs11466045 | 0.004 | VUS |
| FMF | *MEFV* | 10 | p.Ala744Ser | A744S | rs61732874 | 0.002 | VUS |
| FMF | *MEFV* | 2 | p.Arg202Gln | R202Q | rs224222 | 0.136 | benign |
| MKD | *MVK* | 9 | p.Val261Ala | V261A | unknown | unknown | pathogenic |
| MKD | *MVK* | 11 | p.Val377Ile | V377I | rs28934897 | 0.001 | pathogenic |

**Supplemental Table 2**

| **Diagnosis** | **Gene** | **Exon** | **HGVS protein** | **SNP** | **RefSNP Report** | **MAF** | **Function** |
| --- | --- | --- | --- | --- | --- | --- | --- |
| CAPS | *NLRP3* | 3 | p.Arg262Trp | R260W | rs121908150 | unknown | pathogenic |
| CAPS | *NLRP3* | 3 | p.Asp305Asn | D303N | rs121908153 | unknown | pathogenic |
| CAPS | *NLRP3* | 3 | p.Thr350Met | T348M | rs151344629 | unknown | pathogenic |
| CAPS | *NLRP3* | 3 | p.Ala441Val | A439V | rs121908146 | unknown | pathogenic |
| CAPS | *NLRP3* | 3 | p.Gly571Arg | G569R | rs121908151 | unknown | pathogenic |
| CAPS | *NLRP3* | 3 | p.Arg137His | R137H | rs138946894 | 0.0002 | VUS |
| CAPS | *NLRP3* | 3 | p.Val200Met | V198M | rs121908147 | 0.004 | VUS |
| CAPS | *NLRP3* | 3 | p.Leu362Gln | L360Q | rs201593863 | unknown | VUS |
| CAPS | *NLRP3* | 3 | p.Arg490Lys | R488K | rs145268073 | 0.0002 | VUS |
| CAPS | *NLRP3* | 3 | p.Arg556Leu | R554L | unknown | unknown | VUS |
| CAPS | *NLRP3* | 3 | p.Phe581Tyr | F579Y | unknown | unknown | VUS |
| CAPS | *NLRP3* | 3 | p.Pro651Ser | P649S | unknown | unknown | VUS |
| CAPS | *NLRP3* | 3 | p.Gln705Lys | Q703K | rs35829419 | 0.02 | VUS |
| TRAPS | *TNFRSF1A* | 2 | p.Tyr49Cys | Y20C | unknown | unknown | pathogenic |
| TRAPS | *TNFRSF1A* | 2 | p.Tyr49His | Y20H | unknown | unknown | pathogenic |
| TRAPS | *TNFRSF1A* | 3 | p.Cys84Arg | C55R | rs104895253 | unknown | pathogenic |
| TRAPS | *TNFRSF1A* | 3 | p.Thr79Met | T50M | rs104895219 | unknown | pathogenic |
| TRAPS | *TNFRSF1A* | 6 | p.Ile199Asn | I170N | rs104895247 | unknown | pathogenic |
| TRAPS | *TNFRSF1A* | 4 | p.Arg121Gln | R92Q | rs4149584 | 0.006 | VUS |
